# Biopsychosocial Prognosis Scale for Coronary Artery Bypass Grafting – Brazilian Version: Adaptation and Content Validity

**DOI:** 10.21470/1678-9741-2023-0371

**Published:** 2025-02-05

**Authors:** Elisabete Silvana de Oliveira Sene, Renata Eloah de Lucena Ferretti-Rebustini, Meena Hariharan, Marlyn Thomas Savio, Vinicius Batista Santos, Camila Takáo Lopes

**Affiliations:** 1 Escola Paulista de Enfermagem, Universidade Federal de São Paulo, São Paulo, São Paulo, Brazil; 2 Instituto Dante Pazzanese de Cardiologia, São Paulo, São Paulo, Brazil; 3 Escola de Enfermagem, Universidade de São Paulo, São Paulo, São Paulo, Brazil; 4 Center for Health Psychology, University of Hyderabad, Hyderabad, India

**Keywords:** Cross-Cultural Comparasion, Prognosis, Psychometry, Coronary Artery Bypass, Consensus

## Abstract

**Introduction:**

The Biopsychosocial Prognosis Scale for Coronary Artery Bypass Grafting (BIPROSCAB) assesses biophysical symptoms and psychosocial experiences following coronary artery bypass grafting (CABG), thereby enabling the targeting of interventions to improve post-procedure biopsychosocial prognosis. The aim of this study was to adapt the BIPROSCAB for use in Brazil and assess the content validity of the adapted version.

**Methods:**

For the cross-cultural adaptation, English-Portuguese translations, synthesis of translations, back-translations, assessment of back-translations for conceptual consistency by the authors of the original instrument, and evaluation of semantic, idiomatic, cultural, and conceptual equivalences by 11 expert judges were performed. Modifications were made based on suggestions until consensus > 80% was achieved. For the content validity assessment, experts assessed the clarity, theoretical relevance, and practical pertinence of the items, which were considered adequate when the content validity ratio (CVR) > 0.635. Post-CABG patients completed the questionnaire and evaluated understandability of the items.

**Results:**

Three rounds were required to achieve the desired agreement in the cross-cultural adaptation process. In the content evaluation by experts, only one round was needed, with CVR > 0.635. Following content evaluation by patients, it was decided to reverse the order of the response scale to an ascending order.

**Conclusion:**

The Brazilian version, BIPROSCAB-Br, is equivalent to the original instrument and has satisfactory evidence of content validity. Additional psychometric assessments are needed for use in Brazil.

## INTRODUCTION

Coronary artery bypass grafting (CABG) is the recommended treatment for many patients experiencing angina to prevent the risk of further myocardial infarctions, improve ventricular function, and protect an ischemic myocardium^[1]^. Between January 2020 and June 2024, 83,269 CABG procedures were performed in public institutions in Brazil, with a mortality rate of 5.84% and an average hospital stay of 12.1 days, resulting in a cost of over 1.5 billion Brazilian reais to public funds^[2]^.

Evidence suggests that CABG results in an increase in the ejection fraction of the left ventricle and improved quality of life^[3,4]^. However, the prospect of undergoing CABG can generate significant emotional stress and strain for patients and their families. In addition to the physical trauma of the surgery, there is also a risk of mortality, which shapes individual and societal perceptions of the potential sequelae ^[5]^.

Therefore, the recovery phase after the procedure has a significant impact on the patient's life, affecting biopsychosocial aspects of health and well-being. Some symptoms, such as angina, difficulty breathing, depression, fatigue, sleep disturbances, and anxiety, can persist for several weeks to months after surgery. A study conducted in Taiwan found that patients experienced these symptoms three months after CABG^[6]^. At 12 months post-surgery, anxious patients have more outpatient visits while depressed patients have significantly lower quality of life, more readmissions, visits to the emergency sector, home healthcare use, and a longer length of stay^[7]^.

Given the various physiological and psychological repercussions, this period demands adaptation to a new life reality, roles within the family and at work, and social reintegration. Depressive symptoms and social variables, such as social support, influence functionality, survival time, and post-CABG mortality^[8,9]^. Some barriers to individuals' engagement in self-care after CABG include uncertainty, fear, cautiousness, lack of motivation, and depression^[10]^.

Despite this scenario, most studies on post-CABG quality of life focus on physical aspects, often neglecting the biopsychosocial and emotional factors reported by patients. The scarcity of research addressing these biopsychosocial elements, rather than just their clinical status, highlights the need for a more holistic assessment of patients. This approach should consider their perceptions and observations of their own health during recovery. It is essential to clearly understand the cost-benefit of surgery, establishing criteria for a more comprehensive preoperative preparation that includes biopsychosocial considerations, such as the high risk of developing anxiety and depression after surgery^[11,12,13,14]^.

In this context, assessing health status of patients following CABG is crucial for supporting interventions and identifying risk factors that may delay their full recovery^[11,12,13,14]^. Indian researchers hve developed a multidimensional self-report instrument in English to assess and measure the biopsychosocial prognostic factors of CABG patients, named the Biopsychosocial Prognosis Scale for Coronary Artery Bypass Grafting (BIPROSCAB). The instrument consists of 25 items that assess the participant's biophysical symptoms (*e.g.*, "I noticed swelling in both my feet") and psychosocial experiences (*e.g.*, "I missed my social life") after surgery. A five-point scale (from 1= very often to 5 = never) is used to evaluate the items, with scores ranging from 25 to 125. A higher score indicates a better biopsychosocial prognosis for the patient after CABG^[15]^.

The 25 items are structured into nine dimensions: Post-CABG affect state (items 9, 16, 20, and 23; *e.g.*, "I felt very sad and low"), Post-CABG anxiety (items 1, 3, 6, 10, and 12; *e.g.*, "I felt my heartbeat going fast"), Post-CABG physical pain (items 11, 19, and 24; *e.g.*, "I had pain in the chest where they had cut for surgery"), Discomfort in surgical sites (items 7, 13, and 21; *e.g.*, "I experienced numbness in the surgical sites in the leg/arm"), Worry about returning to normalcy (items 2 and 25; *e.g.*, "I found some difficulty in walking normally"), Discomfort in the leg (items 5 and 15; *e.g.*, "I noticed swelling in both my feet"), CABG bio-social by-products (items 4 and 22; *e.g.*, "My family/friends put restrictions on me because of surgery"), Constraints in socializing (items 14 and 18; *e.g.*, "I missed my social life"), and Infection and interference with routine life (items 8 and 17; *e.g.*, "It was strenuous to bathe or dress myself")^[15]^. The BIPROSCAB has demonstrated reliability and validity in studies assessing the impact of clinical and psychosocial interventions on patients after CABG^[15,16]^. Therefore, it is important to adapt this instrument for use in Brazil and evaluate its validity, given the lack of available tools to assess biopsychosocial factors following CABG in the country. The aim of this study was to adapt the BIPROSCAB for use in Brazil and assess the content validity of the adapted version.

## METHODS

### Study Type

This is a psychometric cross-cultural adaptation and content validity assessment of BIPROSCAB for use in Brazil, conducted from April 2021 to January 2022. Authorization was obtained from the original authors. The cross-cultural adaptation (Stage A, Steps A1 to A5) was conducted following Beaton's method^[17]^, with an additional evaluation of the back-translations by the authors of the original BIPROSCAB. Content validity assessment (Stage B) was carried out as proposed by Alexandre et al.^[18]^.

### Stage A) Cross-Cultural Adaptation

**Step A1.** Translation: Two Brazilian translators with proficiency in English independently translated the instrument, producing translations T1 and T2. The first was a professional translator with no experience in the healthcare field, while the second translator was a nurse with a Ph.D.

**Step A2.** Synthesis of T1 and T2: T1 and T2 were synthesized by a third translator (a Brazilian nurse proficient in English, specialized in Cardiology, holding a Ph.D), producing version ST-12.

**Step A3.** Back-translations of ST-12: Two professional translators (native English speakers fluent in Brazilian Portuguese, living in Brazil, with no experience in the healthcare field) independently back-translated version ST1-2 into English, producing versions BT-1 and BT-2.

**Step A4.** Review of BT-1 and BT-2 by the original BIPROSCAB authors: To verify whether ST1-2 was equivalent to the original BIPROSCAB, BT-1 and BT-2 were shared with the authors of the original instrument. The authors were asked to identify potential inconsistencies that would lead to a revision of versions T1 and T2.

**Step A5.** Assessment of linguistic equivalences by a panel of experts: A search for potential experts was carried out on the Lattes Platform of the Brazilian National Council for Scientific and Technological Development (Conselho Nacional de Desenvolvimento Científico e Tecnológico or CNPQ), using the keywords “myocardial revascularization” and “prognosis”, filtering for nurses with Ph.D. Additionally, cardiovascular surgeons and a Portuguese language specialist were invited.

The panel consisted of 11 experts: one physician with a Ph.D. in Cardiovascular Surgery, six nurses with a Ph.D. in Cardiology, one nurse with a post-doctorate in Psychometrics, one Linguistic professional specialized in Portuguese, and the two translators from Steps A1 and A3. The experts received an instrument to evaluate whether the items in ST-12 were equivalent to the original BIPROSCAB using the Delphi technique. Each round of evaluation had a 15-day deadline.

Experts were asked to assess equivalence on a scale where: -1 = not equivalent, 0 = undecided, and +1 = equivalent. If an expert considered any item as not equivalent or undecided, suggestions for changes were requested. Four types of linguistic equivalences were assessed: semantic equivalence (words have the same meaning, and possible grammatical changes in translation were considered), idiomatic equivalence (translated idiomatic and colloquial expressions have the same meaning in the local cultural context), cultural equivalence (translated items were considered in relation to the life experiences of the Brazilian population), and conceptual equivalence (the meaning is representative of the concept of interest)^[19]^.

The percentage of agreement among experts was assessed using the formula: number of participants who scored +1 divided by the total number of judges multiplied by 100. Items with < 80% agreement were re-assessed by the researchers, taking into account the comments and suggestions of the experts, and resubmitted for analysis until achieving adequate agreement^[18]^.

### Stage B) Evaluation of Content Validity of the Adapted Version of BIPROSCAB

Content validity was assessed by two groups: professional experts and post-CABG patients.

**Step B1.** Assessment of content validity by professional experts: the same experts from Stage A, Step A5, individually evaluated the items of ST-12 in terms of clarity (whether the item was drafted in a way that the concept is understandable and adequately expresses what is expected to be measured), theoretical relevance (whether the item demonstrates the cognitive processes of interest), and practical pertinence (whether the item reflects the involved concepts, is relevant, and suitable for achieving the proposed objectives)^[18]^. Evaluations were conducted on a 4-point Likert scale: 1 = not clear/not relevant/not pertinent, 2 = somewhat clear/somewhat relevant/somewhat pertinent, 3 = quite clear/quite relevant/quite pertinent, 4 = very clear/very relevant/very pertinent^[19]^. For each evaluated item, the content validity ratio (CVR) was calculated using the formula:

CVR=[(ne-(N/2))/(N/2)]

Where: ne = number of experts who scored 4, and N = number of experts.

CVR values were considered appropriate based on the number of experts, following Ayre & Scally's recommendations. For 11 judges, the authors determine that a CVR of 0.636 is necessary to achieve a one-tailed *P*-value of 0.033^[20]^. Suggestions were evaluated by the researchers, and items were modified based on suggestions and subjected to new rounds of assessment until satisfactory CVR values were obtained.

**Step B2.** Assessment of content validity by post-coronary CABG patients: the version of BIPROSCAB validated by the group of professional experts was formatted in Calibri font size 14 and administered to 40 patients aged ≥ 18 years who had undergone isolated elective CABG and were able to read and write in Portuguese. Potential participants were approached by the principal investigator during their first postoperative follow-up visit to the Coronary Outpatient Clinic of a public hospital in the state of São Paulo, which typically occurs around 30 days after hospital discharge. Clinical characteristics were collected from their records.

Patient performance in responding to BIPROSCAB was analyzed descriptively. Participants were also interviewed regarding the visual appearance of the instrument (structure and organization of items), font size adequacy, and understanding of instructions and items. Response patterns to the items were assessed by the researchers.

Patient data were entered and analyzed using Microsoft Office Excel 16.3. Categorical variables were expressed as absolute and relative frequencies (n, %). Continuous variables were presented as mean ± standard deviation or median (1st quartile - 3rd quartile). The project was approved by the Research Ethics Committees of the Universidade Federal de São Paulo (Process no. 3.735.512) and the Instituto Dante Pazzanese de Cardiologia (Process no. 3.912.299). All participants signed consent forms.

## RESULTS

### Cross-Cultural Adaptation

In Step A1, Translation, there were word selection discrepancies in Portuguese between the translators, but no semantic differences. In Step A2, the synthesis of translations favored terms that maintained consistency with the original version while facilitating comprehension by a population with lower educational levels. For example, item 17, "Eu desenvolvi infecção no tórax onde cortaram para propósito da cirurgia." was synthesized as "Eu desenvolvi uma infecção no peito onde fizeram o corte para a cirurgia.", and item 12, "Eu tive dificuldade em adormecer." was synthesized as "Eu tive dificuldade em cair no sono."

In Step A3, Back-translations, there were no significant discrepancies between the back-translators, and in Step A4, the authors of the original BIPROSCAB considered that the synthesized version maintained consistency with the original.

In Step A5, Assessment of Linguistic Equivalences by a Panel of Experts, in the first round of evaluation, item 12 had an agreement rate < 80% for idiomatic and cultural equivalences and was modified based on suggestions (Supplementary Table 1). Although item 19 did not have an agreement rate < 80% for any type of equivalence, the modification suggestions were considered relevant by the researchers and accepted (Supplementary Table 1).

**Supplementary Table 1.** Analysis of linguistic equivalences of the Brazilian adapted version of the Biopsychosocial Prognosis Scale for Coronary Artery Bypass Grafting (n=11 experts).

Translated unit	1^st^ round				2^nd^ round				3^rd^ round			
	Sem.	Idiom.	Cult.	Conc.	Sem.	Idiom.	Cult.	Conc.	Sem.	Idiom.	Cult.	Conc.
Instructions and response scale	91.7	91.7	91.7	91.7	-	-	-	-	-	-	-	-
Item 1	100.0	100.0	100.0	100.0	-	-	-	-	-	-	-	-
Item 2	100.0	100.0	100.0	100.0	-	-	-	-	-	-	-	-
Item 3	100.0	100.0	100.0	100.0	-	-	-	-	-	-	-	-
Item 4	91.7	100.0	100.0	100.0	-	-	-	-	-	-	-	-
Item 5	100.0	91.7	100.0	100.0	-	-	-	-	-	-	-	-
Item 6	91.7	100.0	100.0	100.0	-	-	-	-	-	-	-	-
Item 7	100.0	100.0	83.3	100.0	-	-	-	-	-	-	-	-
Item 8	91.7	91.7	100.0	100.0	-	-	-	-	-	-	-	-
Item 9	100.0	100.0	100.0	100.0	-	-	-	-	-	-	-	-
Item 10	91.7	91.7	100.0	100.0	-	-	-	-	-	-	-	-
Item 11	100.0	100.0	100.0	100.0	-	-	-	-	-	-	-	-
Item 12	83.3	75.0	58.3	83.3	100.0	100.0	100.0	100.0	-	-	-	-
Item 13	100.0	100.0	100.0	100.0	-	-	-	-	-	-	-	-
Item 14	91.7	91.7	91.7	91.7	-	-	-	-	-	-	-	-
Item 15	100.0	100.0	100.0	100.0	-	-	-	-	-	-	-	-
Item 16	100.0	91.7	100.0	100.0	-	-	-	-	-	-	-	-
Item 17	100.0	100.0	100.0	100.0	-	-	-	-	-	-	-	-
Item 18	91.7	100.0	100.0	100.0	-	-	-	-	-	-	-	-
Item 19	91.7	91.7	83.3	91.7	75.0	100.0	100.0	100.0	91.7	100.0	100.0	100.0
Item 20	100.0	100.0	100.0	100.0	-	-	-	-	-	-	-	-
Item 21	83.3	91.7	91.7	91.7	-	-	-	-	-	-	-	-
Item 22	100.0	100.0	100.0	100.0	-	-	-	-	-	-	-	-
Item 23	100.0	100.0	100.0	100.0	-	-	-	-	-	-	-	-
Item 24	100.0	100.0	100.0	100.0	-	-	-	-	-	-	-	-
Item 25	100.0	100.0	100.0	100.0	-	-	-	-	-	-	-	-

Conc=conceptual; Cult.=cultural; Idiom.=idiomatic; Sem.=semantic

Therefore, two items (12 and 19) were subjected to a second round of assessment of linguistic equivalences. In the third and final equivalence analysis, item 19 achieved an agreement rate > 80% (Supplementary Table 1). A detailed account of the suggestions made by the experts that were accepted by the researchers is provided in Supplementary Table 2.

**Supplementary Table 2.** Suggestions from the judges that were accepted by the researchers.

Original item and evaluation round	Expert’s suggestion
Item 12. 1^st^ round of assessment: Eu tive dificuldade em pegar no sono	Eu tive dificuldade em cair no sono
Item 19. 1^st^ round of assessment: Eu senti dor em outras áreas do corpo (p.ex.: mãos, costas, ombros, etc)	Eu senti dor em outras partes do corpo (por exemplo: mãos, costas, ombros, etc)
Item 19. 2^nd^ round of assessment: Eu senti dor em outras partes do corpo (por exemplo: mãos, costas, ombros, etc)	Eu tive dor em outras áreas do corpo (por exemplo: mãos, costas, ombros, etc)

### Content Validity

After the equivalence analysis, only one round of content assessment by the same group of experts was necessary for clarity, theoretical relevance, and practical pertinence to be considered adequate by the experts, with CVR > 0.636 for all items (Table 1).

**Table 1.** Content validity ratio per item and overall for the Brazilian adapted version of the Biopsychosocial Prognosis Scale for Coronary Artery Bypass Grafting (n=11 experts).

	Clarity	Practical pertinence	Theoretical relevance
Instructions and response scale	1.000	1.000	1.000
Item 1	1.000	1.000	1.000
Item 2	0.818	1.000	1.000
Item 3	1.000	1.000	1.000
Item 4	1.000	0.818	0.818
Item 5	1.000	1.000	1.000
Item 6	1.000	1.000	1.000
Item 7	0.818	1.000	1.000
Item 8	1.000	1.000	1.000
Item 9	0.818	1.000	1.000
Item 10	1.000	1.000	1.000
Item 11	1.000	1.000	1.000
Item 12	0.636	1.000	1.000
Item 13	1.000	1.000	1.000
Item 14	0.818	1.000	1.000
Item 15	1.000	1.000	1.000
Item 16	0.818	1.000	1.000
Item 17	1.000	1.000	1.000
Item 18	0.818	1.000	1.000
Item 19	1.000	1.000	1.000
Item 20	1.000	1.000	1.000
Item 21	0.818	1.000	1.000
Item 22	1.000	1.000	1.000
Item 23	0.818	1.000	1.000
Item 24	1.000	1.000	1.000
Item 25	1.000	1.000	1.000

### Content Assessment by Patients

Forty patients were invited to participate in the study, and all accepted. Patient characteristics are presented in Table 2. Most patients were elderly, male, active in the job market, had incomplete elementary education, of mixed ethnicity, hypertensive, and received outpatient care from the Central de Regulação de Ofertas de Serviços de Saúde of the State Health Department.

**Table 2.** Sociodemographic and clinical characteristics of postoperative patients after coronary artery bypass grafting (n=40).

Characteristics	Measurement
Age (years), mean ± standard deviation	61 ± 10.68
Male sex, n (%)	30 (75.0)
Active in the job market, n (%)	28 (70.0)
Education, n (%)	3 (8.1)
No education	
Complete primary education	5 (13.5)
Incomplete primary education	13 (31.5)
Complete secondary education	10 (27.0)
Incomplete secondary education	5 (13.5)
Graduated	1 (2.7)
Marital status, n (%)	
Married	26 (63.4)
Single	10 (24.4)
Widower	3 (7.3)
Others	2 (4.9)
Ethnicity, n (%)	
Brown	19 (47.5)
White	16 (40.0)
Black	5 (12.5)
Type of graft, n (%)	
Saphena + mammary	38 (95.0)
Mammary	2 (5.0)
Number of anastomoses, n (%)	5 (12.5)
4	24 (60.0)
2	9 (22.5)
1	2 (5.0)
Comorbidities, n (%)	
Hypertension	39 (41.9)
Diabetes mellitus	18 (19.4)
Dyslipidemia	36 (38.7)
Smoking	12 (30.0)
Former smoking	14 (35.0)
Alcoholic	19 (47.5)
Former alcoholic	9 (22.5)

The response scale originally configured in BIPROSCAB has a descending order of symptom frequency (1 = very often, 2 = 4-5 times, 3 = 2-3 times, 4 = only once, and 5 = never). However, as mentioned by the participants, with support from the literature^[21]^, this configuration requires greater cognitive effort for a response, which can lead to an inaccurate representation of the attribute being measured. Ascending response scales are more intuitive, as respondents tend to associate higher values with a greater intensity or quantity of the attribute being measured^[21]^. Therefore, in the Brazilian version, it was decided to reverse the order of the response scale to "1 = never, 2 = only once, 3 = 2-3 times, 4 = 4-5 times, and 5 = very often". Thus, the lower the score, the better the biopsychosocial prognosis.

There were no suggestions or complaints regarding font size, type, and formatting. The performance of patients in responding to BIPROSCAB - Brazilian Version (BIPROSCAB-Br) is presented in Table 3. The average score of the participants was 49.7, with a standard deviation of 17.1.

**Table 3.** Performance of patients undergoing coronary artery bypass grafting (CABG) when responding to the Biopsychosocial Prognosis Scale for Coronary Artery Bypass Grafting - Brazilian Version (n=40).

Dimensions	Minimum-maximum possible scores*	Mean	Standard deviation
Post-CABG affect state	4-20	8.80	4.36
Post-CABG anxiety	5.-25	9.78	4.31
Post-CABG physical pain	3-15	4.30	2.13
Discomfort in surgical sites	3-15	6.48	3.52
Worry about return to normalcy	2-10	4.10	2.06
Discomfort in the leg	2-10	3.95	2.25
CABG bio-social by-products	2-10	4.43	2.06
Constraints in socializing	2-10	3.23	1.97
Infection and interference to routine life	2-10	2.93	1.37

* The lower the score, the better the prognosis

**Supplementary Fig. 1:**
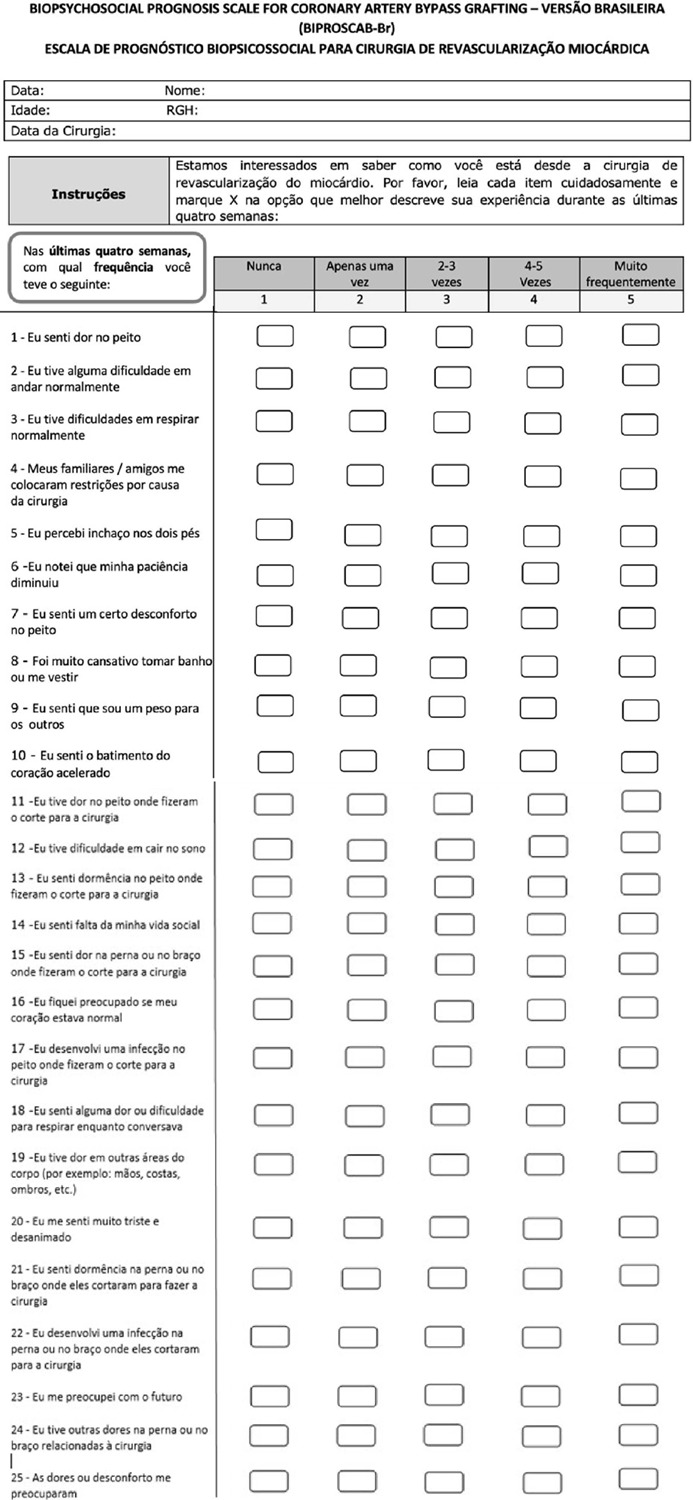
Biopsychosocial Prognosis Scale for Coronary Artery Bypass Grafting – Brazilian version (BIPROSCAB-Br).

The domains that stood out with a better response pattern considering their possible minimum and maximum scores (lower scores, representing a better prognosis) were: Infection and interference in daily life, Socialization limitation, and Physical pain. The items with the highest score (representing a worse prognosis) were Post-CABG affect state, Post-CABG anxiety, and Discomfort in surgical sites. The final version of BIPROSCAB-Br is presented in Supplementary Figure 1.

## DISCUSSION

In this study, an instrument designed to assess the biopsychosocial prognosis 30 days after CABG — the BIPROSCAB — was adapted to the Brazilian culture and had satisfactory evidence of content validity analyzed by a panel of professionals and patients.

The post-CABG patients’ experience interferes with various aspects of their life, with possible implications that require adaptations for a better quality of life. Biophysical symptoms, such as pain and numbness at the incision site, are anticipated consequences in the postoperative recovery process of CABG. However, in some cases, it leads to a decline in physical and emotional functioning if there is no preoperative preparation with education and postoperative rehabilitation follow-up^[22]^.

Cognitive impairment or decline following CABG is prevalent in up to 43% of patients up to four days and remains high (39%) up to one month post-CABG^[23]^. Furthermore, negative feelings lead to psychological changes, including sleep disorders, depression, and anxiety, often persisting for longer periods, ranging from six months to two years, resulting in a negative impact on the post-CABG prognosis^[24,25,26]^. High levels of preoperative anxiety are also associated with depression, leading to a slower recovery prognosis, adverse effects on social and familial relationships, and a heightened sense of pain^[27,28]^. Therefore, it is essential to assess and monitor the patient's psychological state, not just their biophysical state.

The processes of cross-cultural adaptation and validity assessment support specific local practices and the comparison of outcomes and health states among people from different countries. Regarding the content validity in this study, a single round of expert assessment was conducted, achieving an adequate CVR > 0.636 for the adapted Brazilian version. This finding supports an adapted version with language that is understandable to the Brazilian population. Subsequently, content validity of the instrument was considered by the target population, consisting of 40 patients. Educational backgrounds varied, with incomplete primary education predominating, which supports the interpretation that the instrument can be applied across different educational strata of the population.

In this preliminary performance testing of Brazilian patients, Post-CABG affect state, Post-CABG anxiety, and Discomfort in surgical site stood out as the dimensions with the worst prognosis. Interestingly, in the Indian study^[15]^, the dimension of Discomfort at surgical sites was highlighted, which refers to discomfort in the chest, numbness at the surgical incision site on the chest, and numbness at the surgical incision sites on the arm or leg. The Physical pain dimension had the best results in both countries.

Thus, the relevance of a differential investigation of the phenomenon in different populations is confirmed, since Brazilian and Indian patients seem to be facing challenges of Discomfort at the surgical site and Physical pain in similar ways. However, while Infection and interference in daily life dimension stood out with a worse prognosis in India^[15]^, in Brazil, this dimension stood out as one with a better prognosis. Prognostic scores in the dimensions of Post-CABG affect state and Post-CABG anxiety in the Brazilian sample were among the worst, while in the Indian population, they were among the best. For Brazilian patients, issues related to Post-CABG affect (feeling like a burden to others, worrying about the heart being normal, feeling very sad and disheartened, and concerns about the future) and Post-CABG anxiety (feeling chest pain *versus* having pain at the surgical incision site, difficulty breathing normally, decreased patience, accelerated heartbeat, and difficulty falling asleep) stand out. These comparisons suggest that factors such as physiology, culture, and healthcare can influence the postoperative experiences of patients in different regions of the world.

The difference in the dimensions of Post-CABG physical pain and Discomfort at surgical sites between the Brazilian and Indian populations underscores the importance of considering the specific characteristics of each group when planning medical interventions and rehabilitation programs. This includes the development of more personalized postoperative care protocols to meet the distinct needs of each population. The results also emphasize the need for further research to fully understand the reasons behind these differences and to determine how healthcare professionals can adapt their approaches to improve the quality of life and prognosis of patients in different cultural and geographical contexts.

The post-surgical cardiac rehabilitation strategy has been crucial in the recovery process of patients after CABG^[29,30,31]^. Pre- and post-CABG interventions addressing the biophysical, psychological, and social concerns of patients can significantly contribute to the CABG prognosis^[15,16,32]^. The reduction of psychological suffering through psychoeducational intervention generates positive emotions, allowing patients to actively manage their health^[16,33]^.

### Clinical Implications

The BIPROSCAB not only provides a comprehensive view of post-CABG experiences but also potentially allows for the development of personalized care plans that address the specific psychosocial, bio-social, and bio-physiological aspects identified. By understanding the patient's perspective on various dimensions, clinicians can engage in more meaningful and targeted discussions, fostering effective communication and shared decision-making, ultimately leading to improved outcomes and enhanced quality of life^[21,24,25,27,28,29,30,31,32,33]^.

The identification of psychosocial factors (Post-CABG affect state, Post-CABG anxiety, Worry about return to normalcy, and Constraints in socializing) underscores the emotional and social dimensions of a patient's recovery journey. Recognizing aspects such as psychological state, anxiety levels, concerns about returning to normal life, and social limitations can drive the development of interventions supporting emotional well-being and facilitating smoother social reintegration^[21,24,25,27,28,29,30,31,32,33]^.

Addressing bio-social factors (CABG bio-social by-products, Infection and interference to routine life) demands a comprehensive care approach that extends to the broader implications of CABG, including its impact on a patient's social life and daily routines. Implementing strategies to assist patients and their families in adapting to these changes, managing stressors, and maintaining social connections may enhance overall recovery^[21,24,25,27,28,29,30,31,32,33]^.

The acknowledgment of purely bio-physiological factors (Post-CABG physical pain, Discomfort in surgical sites, and Discomfort in the leg) underscores the importance of managing physical symptoms directly linked to the surgical procedure. This information can guide clinicians in developing targeted strategies for pain management and ensuring physical comfort during the recovery process. The presence of post-CABG physical pain and discomfort in surgical sites requires tailored pain management strategies, considering the individual's pain threshold and overall health status. Close monitoring of discomfort in surgical sites goes beyond routine care, requiring additional attention, alongside patient education regarding self-monitoring and decision-making regarding complications^[21,24,25,27,28,29,30,31,32,33]^.

### Limitations

This study should be considered in light of some limitations. The instrument's content was evaluated in only one specialized healthcare center, which may not represent the experience of most CABG patients in Brazil. In this initial analysis, tests have not yet been conducted with patients from different regions and contexts.

## CONCLUSION

The BIPROSCAB-Br is linguistically equivalent to the original instrument and gathers satisfactory evidence of content validity. Additional psychometric evaluations should be carried out, with patient samples that are representative of the Brazilian territory, including evaluation of internal structural validity and relationship with other variables, such as predictive value for return to normalcy. Using the instrument postoperatively as a structured and comprehensive approach may encourage open communication between patients and healthcare professionals, where patients can express their concerns, contributing to a deeper understanding of the patient's post-CABG needs and inclusion of family members in postoperative care.

The instrument may foster better communication among healthcare professionals by providing a shared framework for understanding patient needs. Clear documentation of psychosocial, bio-social, and bio-physiological factors may facilitate communication across disciplines, reducing the risk of information silos, enhancing multidisciplinary collaboration, and enabling teams to allocate resources more efficiently.

## Figures and Tables

**Referência original:** Hariharan M. Thorras M. Rana S. Development and Preliminary Testing of the Biopsychosocial Prognosis Scale for Coronary Artery Bypass Grafting. *Indian J Psychol Med.* 2017;39(5):619-626. doi:10.4103/IJPSYM.IJPSYM_124_17

## Abbreviations, Acronyms & Symbols

BIPROSCAB: Biopsychosocial Prognosis Scale for Coronary Artery Bypass Grafting
CABG: Coronary artery bypass grafting
Conc.: Conceptual
Cult.: Cultural
CVR: Content validity ratio
Idiom.: Idiomatic
Sem.: Semantic

## Appendix

A BIPROSCAB foi construída para mensurar, a partir de uma perspectiva biopsicossocial, o prognóstico de pacientes submetidos à cirurgia de revascularização do miocárdio (CRVM). A avaliação quantifica o prognóstico do paciente em termos de nível de bem-estar após a CRVM. A BIPROSCAB possui 25 itens, mensurados por meio de uma escala de 5 pontos, variando em termos de frequência (1 = Muito frequentemente, 5 = Nunca).

***Pontuação.*** A pontuação total do prognóstico é obtida pela soma das pontuações em todos os itens. Essa pontuação total varia entre 25 e 125. Na versão brasileira, quanto menor a pontuação, melhor é o prognóstico total. O padrão de pontuação das dimensões é apresentado abaixo. Uma menor pontuação em uma dimensão implica em melhor prognóstico nessa dimensão.

**Table:**

Dimensão	Itens	Variação da pontuação
1 Estado do afeto pós CRVM 2 Ansiedade pós CRVM 3 Dor física pós CRVM 4 Desconforto em sítios cirúrgicos 5 Preocupação com o retorno à normalidade 6 Desconforto na perna 7 Subprodutos biossociais da CRVM 8 Limitações na socialização 9 Infecção e interferência na vida cotidiana	9, 16, 20 e 23	4—20
	1, 3, 6, 10 e 12	5—25
	11, 19 e 24	3—15
	7, 13 e 21	3—15
	2 e 25	2—10
	5 e 15	2—10
	4 e 22	2—10
	14 e 18	2—10
	8 e 17	2—10
